# Neuroimaging features of antiphospholipid antibody-related stroke compared with atrial fibrillation-related stroke

**DOI:** 10.1038/s41598-022-16019-3

**Published:** 2022-07-08

**Authors:** Wookjin Yang, Dong-Wan Kang, Jeong-Min Kim, Keun-Hwa Jung, Seung-Hoon Lee

**Affiliations:** 1 Department of Neurology, Seoul National University Hospital, 101 Daehak-ro, Jongno-gu, Seoul, 03080 Korea; 2 Korean Cerebrovascular Research Institute, Seoul, Korea; 3 Cenyx Biotech Inc., Seoul, Korea

**Keywords:** Neurology, Rheumatology, Cerebrovascular disorders, Stroke

## Abstract

Recognizing the lesion pattern of antiphospholipid antibody-related stroke (aPL-stroke) may contribute to establishing the cause in patients with cryptogenic stroke. We aimed to describe the neuroimaging features of aPL-stroke compared with atrial fibrillation-related stroke (AF-stroke), a major hidden cause of cryptogenic stroke. Using a prospective stroke registry, we identified consecutive aPL- and AF-stroke patients without other potential causes of stroke. Neuroimaging features based on diffusion-weighted imaging and angiographic findings at admission were compared. A total of 56 and 333 patients were included in the aPL- and AF-stroke groups, respectively. aPL-stroke patients more often presented with single small lesions (aPL-stroke, 30.4% vs. AF-stroke, 7.5%, *p* < 0.001), while the predominant pattern in AF-stroke patients was large territorial lesions (26.8% vs. 56.5%, *p* < 0.001). aPL-stroke patients had smaller infarct volume (1.58 mL [0.45; 9.41] vs. 11.32 mL [2.82; 33.08], *p* < 0.001) and less experience of relevant artery occlusion (17.9% vs. 54.7%, *p* < 0.001). The proportion of multi-territory lesions, an embolic pattern, was similar between the two groups (28.6% vs. 22.8%, *p* = 0.44). In comparison only including patients with multi-territory lesions as well, aPL-stroke patients showed small lesion dominance and smaller infarct volume. Multivariate analyses showed independent associations between mild neuroimaging features (small lesion prevalence, smaller infarct volume, and absence of relevant artery occlusion) and aPL-stroke. Patterns of small lesion prevalence, small infarct volume, and absence of relevant artery occlusion were suggestive of aPL-stroke rather than AF-stroke. Cryptogenic stroke patients with such neuroimaging features may benefit from aPL testing for a precise diagnosis.

## Introduction

The association between antiphospholipid antibody (aPL) and ischemic stroke was first described some time ago. However, information on the neuroimaging pattern of aPL-related stroke (aPL-stroke) remain scarce. The few studies that have examined this topic have limitations, such as that neuroimaging evaluations were only performed on selected patients rather than on a routine basis, not addressing the potential effect of alternative causes of stroke other than aPL on the neuroimaging pattern, or drawing a conclusion based on analysis of heterogeneous disease groups including cerebral venous thrombosis, seizure, or migraine, as well as cerebral infarction^1–4^. In addition, tremendous advances in diagnostic approaches for stroke and vascular risk factor management have not been reflected in these studies as they were mainly conducted in the 1990s. Thus, the imaging characteristics of aPL-stroke remain to be clarified.

From a neurologist’s perspective, recognizing the characteristics of aPL-stroke may be advantageous for reducing cryptogenic stroke. Great effort has been focused on finding occult atrial fibrillation (AF) in the diagnosis of cryptogenic stroke, and AF is reportedly detected in 25% of patients with cryptogenic stroke^5^. However, the underlying etiologies of cryptogenic stroke can be diverse, and aPL-stroke may also be an underestimated cause. From this point of view, we recently reported a high aPL positivity rate in patients with cryptogenic stroke regardless of age^6^. Identifying distinct features of aPL-stroke compared with AF-related stroke (AF-stroke), the most clinically important cause of cryptogenic stroke, may provide guidance for efficient aPL screening in cryptogenic stroke and subsequently contribute to reduced diagnostic uncertainty. Furthermore, recognizing aPL-stroke may benefit in preventing direct oral anticoagulant (DOAC) misuse in underdiagnosed aPL-stroke patients, considering recent reports of DOAC-related harm in antiphospholipid syndrome (APS)^7–9^. For this reason, we aimed to describe the neuroimaging characteristics of aPL-stroke compared to AF-stroke in the present study.

## Results

**Figure 1:**
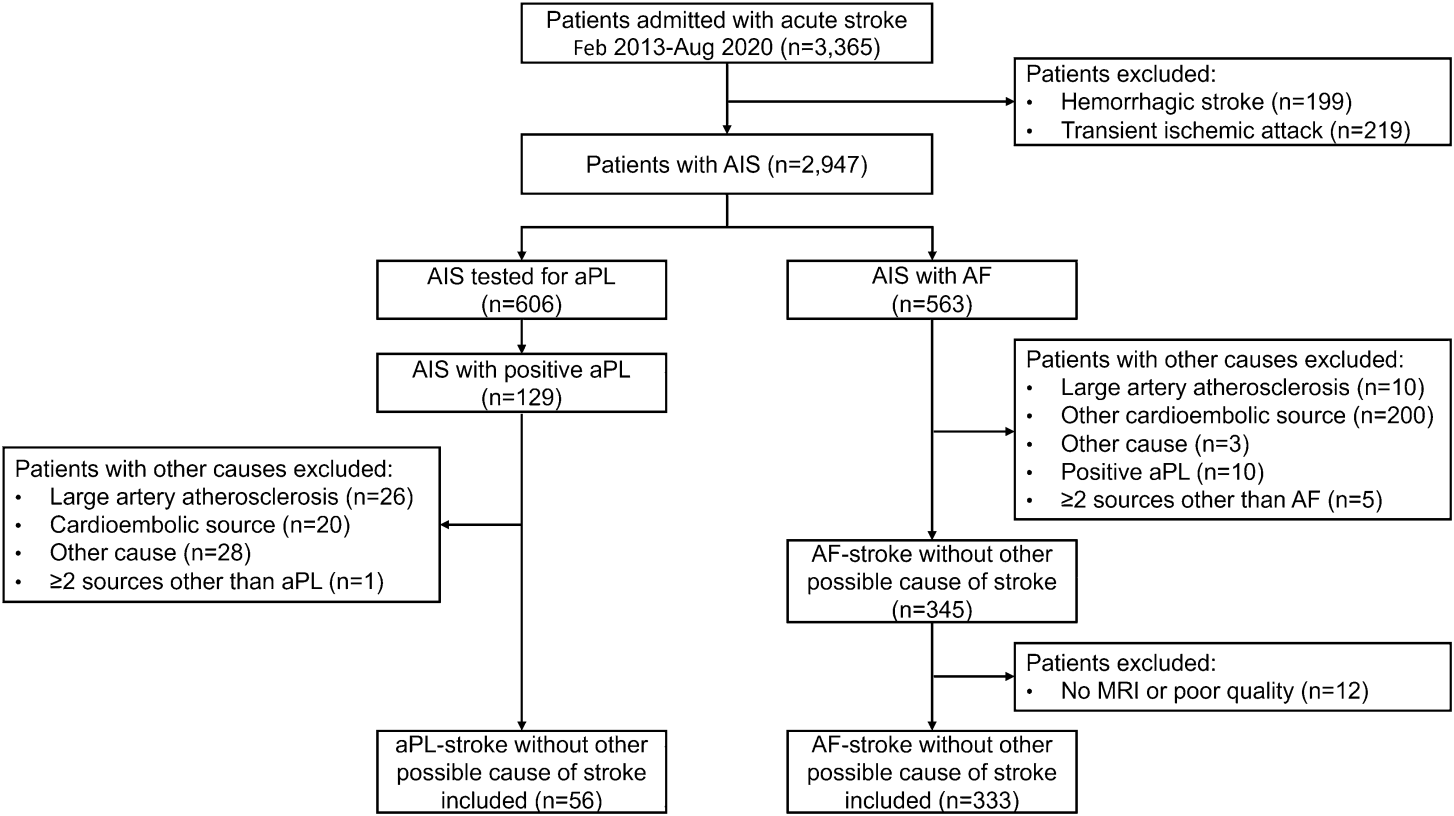
Flowchart of patient inclusion and exclusion. *AF* atrial fibrillation, *AF-stroke* atrial fibrillation-related stroke, *AIS* acute ischemic stroke, *aPL* antiphospholipid antibody, *aPL-stroke* antiphospholipid antibody-related stroke, *MRI* magnetic resonance imaging.

A total of 56 and 333 aPL- and AF-stroke patients were included in the analysis, respectively (Fig. 1). The median [interquartile range] age was 75 [65; 80] years, and 216 (55.5%) were males.

The baseline characteristics are shown in Table 1. aPL-stroke patients were younger than AF-stroke patients. Nevertheless, they were more likely to be smokers, and the proportions of hypertensive, diabetic, and hyperlipidemic patients were comparable between the two groups. aPL-stroke patients were less likely to have a stroke history and to use antithrombotics. The neurological severity was milder, and the need for thrombolytic therapy was less frequent in the aPL-stroke group. Platelet count and low-density lipoprotein (LDL) cholesterol levels were higher in patients with aPL-stroke. AF-stroke patients showed higher fasting glucose levels and prothrombin time-international normalized ratio (PT-INR), however, the absolute differences were not significant. Transthoracic and transesophageal echocardiography were performed in 98.7% (n = 384) and 20.1% (n = 78) of the patients included in the analysis, respectively. The left atrium was significantly enlarged in AF-stroke patients compared to aPL-stroke patients.

**Table 1:** Clinical and laboratory characteristics of aPL- and AF-stroke patients.

	aPL-stroke (n = 56)	AF-stroke (n = 333)	*p-*value
Age, years	61 [48; 68]	76 [68; 81]	< 0.001
Sex, male	27 (48.2)	189 (56.8)	0.30
Body mass index, kg/m^2^	23.7 ± 3.5	23.3 ± 3.4	0.52
Hypertension	39 (69.6)	239 (71.8)	0.87
Diabetes	12 (21.4)	111 (33.3)	0.11
Hyperlipidemia	25 (44.6)	131 (39.3)	0.55
Previous stroke	4 (7.1)	66 (19.8)	0.036
Ever smoker	25 (44.6)	93 (27.9)	0.018
**Previous medication**			
Antiplatelet	11 (19.6)	132 (39.8)	0.006
Anticoagulant	3 (5.4)	65 (19.5)	0.017
**Neurologic status**			
Initial NIHSS score	3 [1; 5.5]	5 [2; 14]	0.003
Discharge NIHSS score	1 [0; 4]	2 [1; 8]	0.015
Discharge mRS	1 [1; 2]	2 [1; 4]	0.003
Intravenous thrombolysis	3 (5.5)	52 (15.6)	0.067
Endovascular treatment	3 (5.5)	63 (18.9)	0.021
White blood cells, × 10^9^/L	8.425 [6.040; 10.875]	7.480 [6.250; 9.410]	0.22
Hematocrit, %	40.6 [36.4; 45.0]	40.0 [35.9; 44.4]	0.50
Platelets, × 10^9^/L	234 [195.5; 268.5]	201 [167; 233]	< 0.001
Creatinine, mg/dL	0.86 [0.70; 0.99]	0.90 [0.74; 1.12]	0.061
Fasting glucose, mg/dL	97.5 [85; 106]	100 [87; 124]	0.034
LDL cholesterol, mg/dL	118 [87.5; 152]	96.5 [71; 125]	0.002
PT-INR	0.97 [0.94; 1.01]	1.03 [0.97; 1.11]	< 0.001
Fibrinogen, mg/dL	334 [293; 384]	320 [280; 372]	0.20
hsCRP, mg/dL	0.15 [0.10; 0.56]	0.23 [0.08; 0.81]	0.38
LVIDd, cm	4.7 [4.5; 5.0]	4.7 [4.4; 5.0]	0.44
LVIDs, cm	2.9 [2.7; 3.1]	3.0 [2.7; 3.2]	0.72
IVSd, cm	0.9 [0.8; 1.0]	1.0 [0.9; 1.1]	0.040
LVPWd, cm	0.9 [0.9; 1.0]	1.0 [0.9; 1.0]	0.14
LV mass index, g/m^2^	89.0 [77.6; 106.2]	93.4 [78.6; 109.0]	0.35
LV ejection fraction, %	61 [57.5; 65]	60 [56; 64]	0.18
Left atrial size, mm	39 [35; 43]	49 [43; 53]	< 0.001
Subclinical valve lesion	8 (14.3)	67 (20.1)	0.40

The data were expressed as numbers (%), mean ± standard deviation, or median (interquartile range).

*AF-stroke* atrial fibrillation-related stroke, *aPL-stroke* antiphospholipid antibody-related stroke, *hsCRP* high-sensitivity C-reactive protein, *IVSd* interventricular septal thickness at end-diastole, *LDL* low-density lipoprotein, *LV* left ventricular, *LVIDd* LV internal diameter at end-diastole, *LVIDs* LV internal diameter at end-systole, *LVPWd* LV posterior wall thickness at end-diastole, *mRS* modified Rankin Scale, *NIHSS* National Institutes of Health Stroke Scale, *PT-INR* prothrombin time-international normalized ratio.

**Figure 2:**
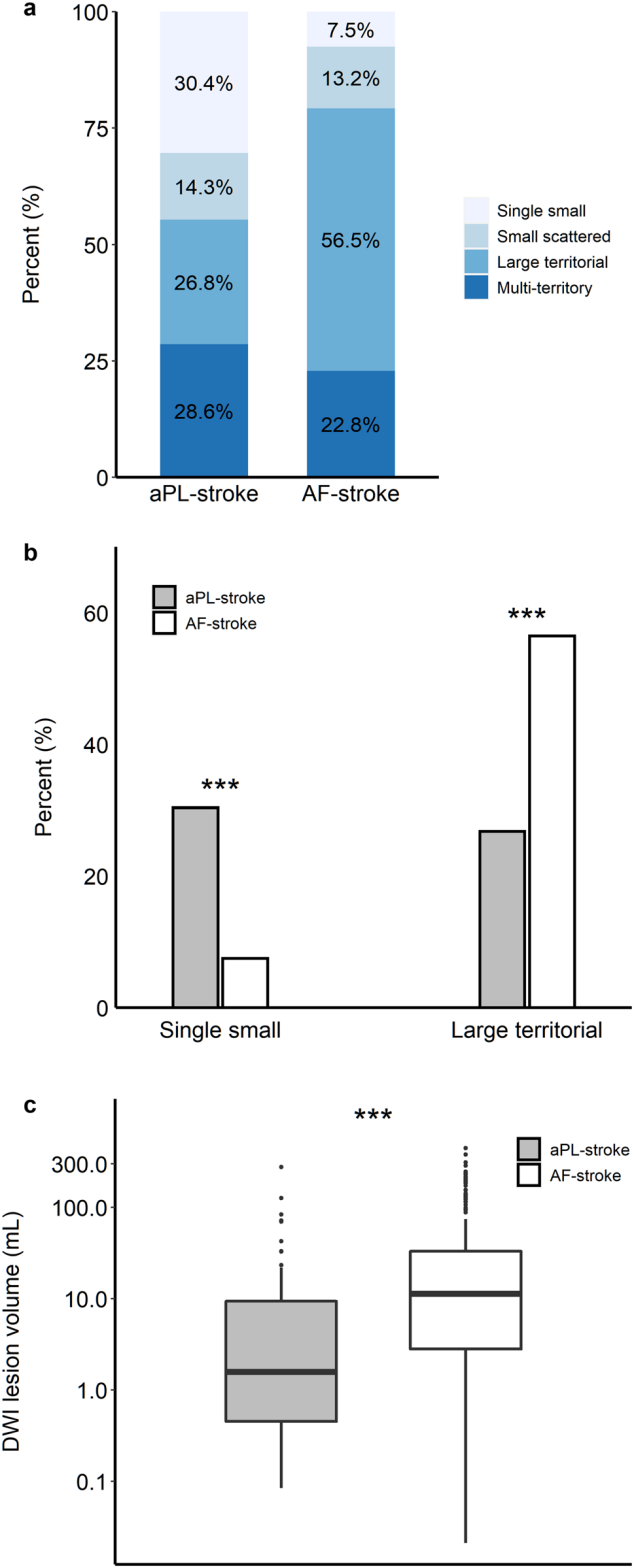
Neuroimaging features of aPL- and AF-stroke patients. (**a**) Lesion pattern based on the size and distribution of aPL- and AF-stroke patients. A single small lesion, solitary lesion ≤ 15 mm; small scattered lesion in a single territory, multiple scattered lesions with the largest lesion size ≤ 15 mm; large territorial lesion, lesion > 15 mm involving single vascular territory; and multi-territory lesion, multiple lesions involving multiple vascular territories. (**b**) Comparison of representative lesion patterns in aPL- and AF-stroke patients. (**c**) Total DWI lesion volumes of aPL- and AF-stroke patients. DWI lesion volume is presented on the y-axis as a log scale. ***p < 0.001. *AF-stroke* atrial fibrillation-related stroke, *aPL-stroke* antiphospholipid antibody-related stroke, *DWI* diffusion-weighted imaging.

**Figure 3:**
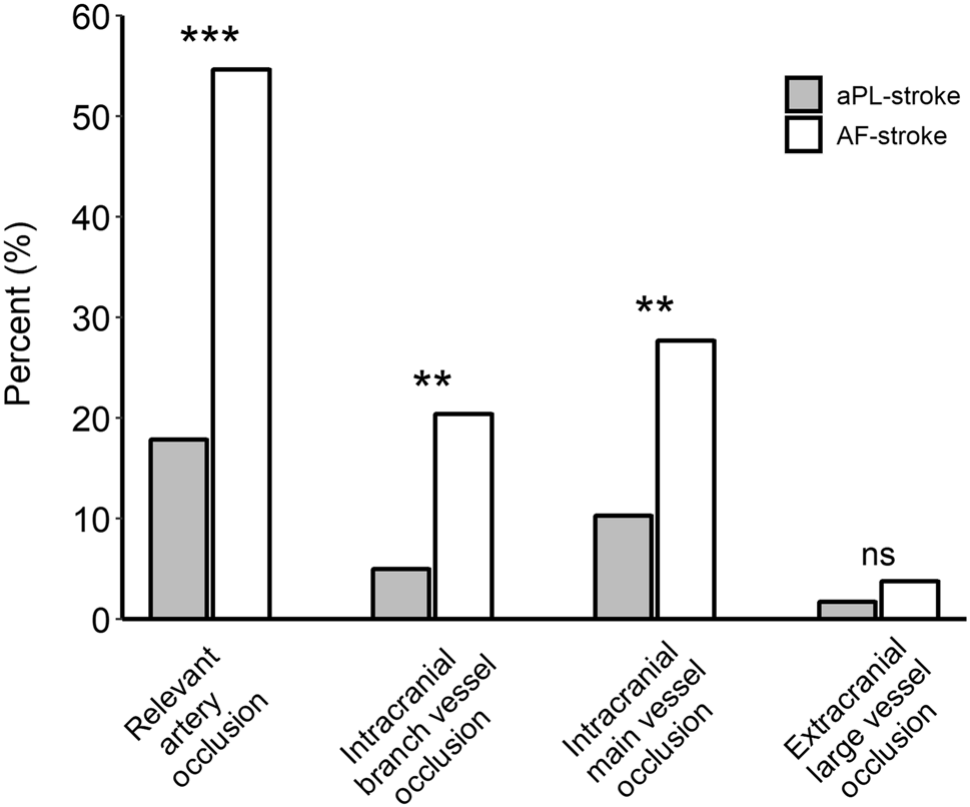
Proportion of patients who experienced relevant artery occlusion among aPL- and AF-stroke patients. Intracranial branch vessel occlusion, occlusion of the ACA, PCA, M2 or distal segments of the MCA, or SCA; intracranial main vessel occlusion, occlusion of the distal ICA, M1 segment of the MCA, distal VA, or BA; and extracranial large vessel occlusion, occlusion of the CCA, proximal ICA, or proximal VA. ***p < 0.001; **p < 0.01; *ns* no significant difference. *ACA* anterior cerebral artery, *AF-stroke* atrial fibrillation-related stroke, *aPL-stroke* antiphospholipid antibody-related stroke, *BA* basilar artery, *CCA* common carotid artery, *ICA* internal carotid artery, *MCA* middle cerebral artery, *PCA* posterior cerebral artery, *SCA* superior cerebellar artery, *VA* vertebral artery.

**Figure 4:**
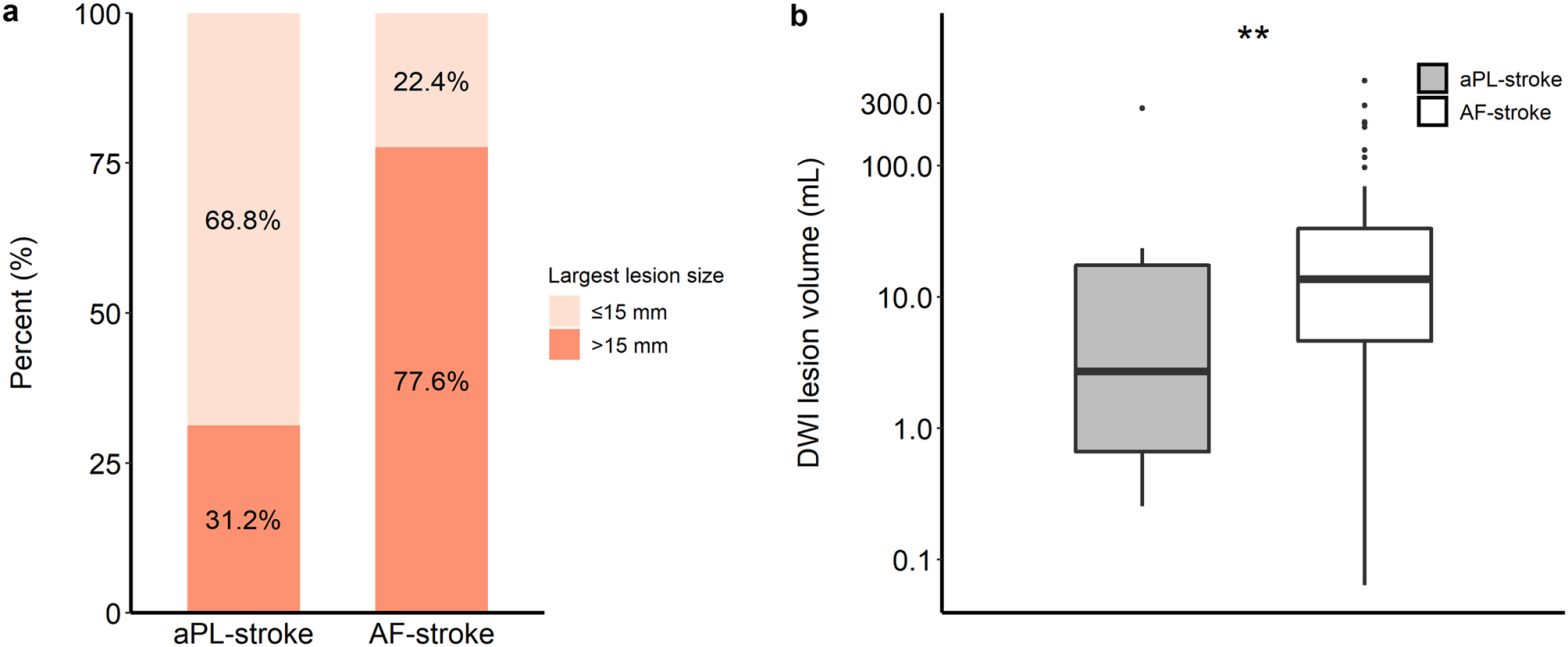
DWI lesion pattern and total lesion volume of aPL- and AF-stroke patients with a multi-territory lesion. (**a**) Lesion pattern based on the largest lesion size (≤ 15 mm or > 15 mm) of aPL- and AF-stroke patients with multi-territory lesions. (**b**) Total DWI lesion volumes of aPL- and AF-stroke patients with multi-territory lesions. DWI lesion volume is presented on the y-axis as a log scale. **p < 0.01. *AF-stroke* atrial fibrillation-related stroke, *aPL-stroke* antiphospholipid antibody-related stroke, *DWI* diffusion-weighted imaging.

More patients presented with a single small lesion in the aPL-stroke group, whereas more than half of the AF-stroke patients had a large territorial infarction (Fig. 2a,b). The total diffusion-weighted imaging (DWI) lesion volume was significantly smaller in aPL-stroke patients (Fig. 2c). Over 80% of aPL-stroke patients had no relevant artery occlusion, while more than half of the AF-stroke patients experienced occlusion of the pertinent artery (Fig. 3). The proportion of multi-territory lesions was similar between the two groups (aPL-stroke, 16 [28.6%]; AF-stroke, 76 [22.8%]; *p* = 0.44). In the analysis that only included patients with multi-territory lesions, aPL-stroke patients tended to have small (≤ 15 mm) scattered lesions, in contrast to AF-stroke patients in which the predominant pattern was confluent (> 15 mm) with additional lesions. The total DWI lesion volume was smaller in aPL-stroke patients than in AF-stroke patients with multi-territory lesions (Fig. 4). In the multivariate analyses, the largest lesion size ≤ 15 mm in diameter, smaller total DWI lesion volume, and absence of relevant artery occlusion were independently associated with aPL-stroke (Table 2).

**Table 2:** Univariate and multivariate binary logistic regression of the neuroimaging parameters in aPL-stroke.

	Univariate	Multivariate	
	OR (95% CI)	Model I^a^	Model II^b^
		OR (95% CI)	OR (95% CI)
Largest lesion size ≤ 15 mm	5.17 (2.84–9.41)	4.83 (2.39–9.76)	5.07 (2.37–10.85)
Smaller infarct volume^c^	1.27 (1.15–1.40)	1.26 (1.12–1.42)	1.28 (1.12–1.45)
Absence of relevant artery occlusion	5.54 (2.71–11.36)	7.43 (3.09–17.85)	6.93 (2.78–17.27)

*aPL-stroke* antiphospholipid antibody-related stroke, *CI* confidence interval, *DWI* diffusion-weighted imaging, *OR* odds ratio.

^a^Adjusted for sex and age.

^b^Adjusted for sex, age, body mass index, hypertension, diabetes, hyperlipidemia, history of previous stroke, and smoking.

^c^OR per twofold decrease in total DWI lesion volume.

Twenty-one patients in the aPL-stroke group had definite APS. Definite APS-stroke patients had comparable clinical, laboratory, and imaging characteristics to those of the aPL-stroke group. The comparison results between the definite APS- and AF-stroke groups were generally in line with those of the above analysis, which compared the aPL- and AF-stroke groups (Tables S1 and S2). Likewise, the infarct burden of patients with multi-territory lesions was lower in the definite APS-stroke group (Fig. S1).

## Discussion

In the present study, neuroimaging patterns of small lesion dominance, smaller total infarct volume, and absence of relevant artery occlusion were associated with aPL-stroke rather than AF-stroke. Although the proportion of multi-territory lesions, which is indicative of embolic infarction, was comparable, the infarct burden of patients with this lesion pattern was lower in aPL-stroke than in AF-stroke. Sensitivity analysis, which compared clinical, laboratory, and imaging characteristics between definite APS- and AF-stroke, showed similar results.

Until now, the underlying mechanism by which aPL precipitates ischemic stroke has not been clearly demonstrated. Accentuation of atherosclerosis and evolution of cardiac problems are suggested to be provoked in the presence of aPL, eventually leading to ischemic stroke^10–12^. In the present study, the proportion of patients presenting with multi-territory lesions among aPL-stroke was comparable to that of AF-associated cardioembolic stroke, making up nearly 30% of cases. Taken together with milder neuroimaging features in aPL-stroke, the findings of our study may suggest that patients with positive aPL may experience ischemic stroke attributed to small-sized emboli originating proximal to large cervical vessels, rather than to direct involvement of intracranial vessels. The sources of such proximal embolism can be cardiac causes or thrombus developed at the wall of the proximal arteries. Most cardioembolic infarctions arise from one of the following processes: thrombus evolution in the left cardiac chambers, embolization of valve-related debris, or paradoxical embolism. Thrombus of the left chamber origin has been reported to be associated with an enlarged left atrium and larger embolus^13^. Thus, it is unlikely that thrombosis occurs in the left chambers in aPL-stroke, which had smaller left atrial size and neuroimaging features suggestive of low embolus burden in our study. Changes in cardiac valves in APS have been suggested to result in stroke in previous studies^12^, however, the proportion of patients with subclinical valve lesions was not as high in aPL-stroke compared with AF-stroke in our study. Patients with intra/extracardiac shunt were excluded from the analysis due to the uncertainty of aPL’s role in their stroke. Rather than cardiac sources, culprit thrombus may grow at the arteries proximal to large cervical vessels. Under the presence of aPL, a higher proportion of smokers, higher LDL level, and similar prevalence of hypertension and hyperlipidemia compared with AF-stroke despite their younger age in aPL-stroke patients may have acted as ‘second hits’ which precipitate thrombus formation at the site of minor endothelial injury^14^.

Another 30% of aPL-stroke patients presented with a single small lesion. This lesion pattern may be caused by either an embolism with small thrombi at the proximal sites or thrombosis directly involving intracranial arteries^15,16^. Different underlying pathophysiologies may need to be considered depending on the lesion pattern in aPL-stroke. Further studies on the mechanism of aPL-stroke based on different lesion patterns would be of value to help enhance secondary prevention.

This study had several limitations. First, tests for aPL were performed at the discretion of physicians rather than in a routine manner in our study and this may have introduced some selection bias. Second, patients with positive aPL and other alternative causes of stroke such as large artery atherosclerosis were excluded. Accordingly, the contribution of aPL-associated changes in systemic vasculature may have been underestimated. Nevertheless, it is not considered important to differentiate whether coexisting atherosclerosis is aPL-associated or not in real-world practice. Given that aPL testing has the highest clinical importance in cryptogenic strokes, such underestimation may not be critical from a practical perspective. Third, the control patients in our study had documented AF, and they may have some differences in clinical manifestation from cryptogenic stroke patients with AF detected after stroke, which are of clinical interest. Furthermore, despite the importance of AF in real-world diagnostic practice for cryptogenic stroke, the AF-stroke group may not fully reflect the spectrum of cryptogenic stroke. Patients classified as stroke of undetermined cause might have been a better control group, however, we decided not to use them as a control since its definition could vary greatly depending on the managing physicians, given the retrospective design and the lack of prespecified workup criteria for defining undetermined stroke in our study. Finally, the results of this study cannot be widely generalized due to its single-center, retrospective design.

We presented distinct neuroimaging features of aPL-stroke from AF-stroke, one of the most important hidden causes of cryptogenic stroke. It would be plausible to perform aPL testing in patients with cryptogenic stroke with low infarct burden and no visible arterial occlusion to provide an additional diagnosis.

## Methods

### Study population

The Seoul National University Hospital (SNUH) Stroke Registry is a prospective stroke registry database that enrolled consecutive patients admitted with acute stroke within seven days to SNUH, one of the largest tertiary referral centers in Korea. Patients who were registered in the SNUH Stroke Registry from 2013 to 2020 were reviewed for eligibility. Patients with hemorrhagic stroke or transient ischemic attack were excluded. Among the acute ischemic stroke cases, those with positive aPL testing results were classified as the aPL-stroke group. Patients with evidence of other potential causes of stroke were excluded. Patients with a previously established diagnosis of AF or newly diagnosed AF at the time of stroke were classified into the AF-stroke group. Among the AF-stroke group, those with potential cardioembolic sources other than AF or noncardiac causes of stroke were excluded in a similar manner as in the aPL-stroke group. A detailed list of the coexisting potential sources that were excluded from the analysis is presented in Table S3. Patients with poor quality or absence of magnetic resonance imaging (MRI) images were also excluded. The institutional review board at the Seoul National University Hospital (H-2002–019-1099) approved this study and waived the need for written informed consent. The present study was performed in accordance with the Declaration of Helsinki.

### Clinical and laboratory data

Sex, age, height, weight, presence of hypertension, diabetes, hyperlipidemia, stroke history, smoking, concurrent antithrombotics, thrombolytic therapy, and the laboratory findings including complete blood count, creatinine, fasting glucose, LDL cholesterol, PT-INR, fibrinogen, and high-sensitivity C-reactive protein were collected at the time of admission. Echocardiographic measurements, including left ventricular internal diameter at end-diastole and end-systole, interventricular septal thickness at end-diastole, left ventricular posterior wall thickness at end-diastole, left ventricular mass indexed to body surface area, left ventricular ejection fraction, and left atrial size were obtained using transthoracic echocardiography. The presence of subclinical valve lesions, defined as thickening, sclerosis, or calcification of the aortic or mitral valve without significant flow disturbance, was collected based on the findings of transthoracic or transesophageal echocardiography. The National Institutes of Health Stroke Scale score at admission and discharge, and the modified Rankin Scale score at discharge were assessed to evaluate neurologic severity.

### Antiphospholipid antibody testing

aPL-stroke patients were tested for aPL using lupus anticoagulant (LA), anti-cardiolipin antibody (aCL) IgG/IgM, and anti-β2-glycoprotein I antibody (β2GPI) IgG/IgM and were positive for at least one aPL. Tests for LA were performed using the ACL TOP 750 analyzer (Instrumental Laboratory, Bedford, MA, USA) according to the recommendations of the International Society on Thrombosis and Haemostasis, through a screening, mixing, and confirmatory test with the diluted Russel Viper venom time and silica clotting time^17^. Tests for aCL and β2GPI were performed using the HemosIL AcuStar testing system (Instrumentation Laboratory, Bedford, MA, USA) according to the manufacturer’s instructions. The normalized screen-to-confirmatory ratios exceeding 1.21 for the diluted Russel Viper venom time and 1.26 for the silica clotting time, respectively, were considered positive for LA. The cut-off values for aCL and β2GPI IgG/IgM were set to 20 U/mL according to the manufacturer’s instructions^18,19^.

### Imaging characteristics

All patients underwent brain MRI including DWI and intra/extracranial angiography with at least one of computed tomography (CT), MR, or conventional angiography, within seven days of onset. The lesion pattern on DWI was classified into four categories based on the size and distribution: single small lesion, a solitary lesion of ≤ 15 mm in maximal diameter; small scattered lesion in a single territory, multiple scattered lesions with the largest lesion size of ≤ 15 mm; large territorial lesion, lesion greater than 15 mm, which involves a single vascular territory; and multi-territory lesion, multiple lesions involving multiple vascular territories (Fig. S2). The vascular territory was categorized based on the distribution of DWI lesions: single territory, lesions located in one of the right internal carotid artery (ICA) territory, left ICA territory, or vertebrobasilar artery territory; multi-territory, lesions scattered in ≥ 2 of the above territories. The total DWI lesion volume was calculated in each case using a semiautomated method based on the 3D slicer (v4.11.2, http://slicer.org), an open-source software (Fig. S3). The presence of relevant artery occlusion was assessed by CT, MR, or conventional angiography. Those with relevant artery occlusion were further classified according to the site of occlusion: intracranial branch vessel occlusion, occlusion of anterior cerebral artery, posterior cerebral artery, M2 or distal segments of the middle cerebral artery (MCA), or superior cerebellar artery; intracranial main vessel occlusion, distal ICA, M1 segment of MCA, distal vertebral artery (VA), or basilar artery; and extracranial large vessel occlusion, common carotid artery (CCA), proximal ICA, or proximal VA (Fig. S4)^20^. All the imaging parameters were assessed by two experienced neurologists (W.Y. and D.-W.K.) blinded to the clinical information. In cases where a disagreement occurred between the two neurologists, a third neurologist (S.-H.L.) was consulted to reach a consensus.

### Statistical methods

Continuous parameters were analyzed using the t-test or Mann–Whitney U test, depending on the normality of the distribution. Categorical parameters were analyzed using the chi-square test or Fisher’s exact test, as appropriate. Among the four categories of lesion patterns in our study, multi-territory lesions were suggestive of embolic source proximal to the CCA and VA origins^21^. Given that all the patients who had scattered lesions in multiple territories were classified into this category regardless of their infarct burden, the size of the largest lesion and total DWI lesion volume were compared between aPL- and AF-stroke patients with multi-territory lesions to examine the difference in infarct burden. To assess the independent relationship between aPL-stroke and neuroimaging parameters (i.e., the largest lesion size ≤ 15 mm, smaller total DWI lesion volume, and the absence of relevant artery occlusion), multivariate binary logistic regressions were conducted for each of the above parameters with adjustment for the following factors: sex, age, body mass index, hypertension, diabetes, hyperlipidemia, stroke history, and smoking. The total DWI lesion volume was logarithmically transformed as it was non-normally distributed. We performed a sensitivity analysis that only included definite APS patients according to the Sydney criteria^22^, classifying them as definite APS-related stroke (APS-stroke) group. Two-sided probability values < 0.05 were considered statistically significant. Statistical analyses were performed using R (v4.1.0, R Foundation, Vienna, Austria).

### Ethical approval

The institutional review board at SNUH (H-2002-019-1099) approved this study and waived the need for written informed consent.

## Supplementary Information

Supplementary Information.

## Data Availability

The data that support the findings of this study are available from the corresponding author upon reasonable request.
