# Supplementary material for: Neuroimaging features of antiphospholipid antibody-related stroke compared with atrial fibrillation-related stroke
Source: Sci Rep. 2022 Jul 8;12:11686. doi: 10.1038/s41598-022-16019-3 (PMC9270426; doi:10.1038/s41598-022-16019-3)
Supplement: Supplementary file 1 — Supplementary Information. [file 41598_2022_16019_MOESM1_ESM.pdf]

ONLINE SUPPLEMENTAL MATERIAL:

Neuroimaging features of antiphospholipid antibody-related stroke compared with atrial fibrillation-related stroke

Wookjin Yang, MD, MSc[1, 2]; Dong-Wan Kang, MD, MSc[1, 2]; Jeong-Min Kim, MD, PhD[1]; Keun-Hwa Jung, MD, PhD[1]; Seung-Hoon Lee, MD, PhD, FAHA[1, 2, 3]

[1] Department of Neurology, Seoul National University Hospital, Seoul, Korea

[2] Korean Cerebrovascular Research Institute, Seoul, Korea

[3] Cenyx Biotech Inc., Seoul, Korea

Table S1. Baseline clinical characteristics of the definite APS-stroke, aPL-stroke, and AF-stroke groups

	**Definite APS-stroke (n=21)**	**aPL-stroke (n=56)**	**AF-stroke (n=333)**	***p*1**	***p*2**
Age, years	56 [45; 64]	61 [48; 68]	76 [68; 81]	0.14	<0.001
Sex, male	9 (42.9)	27 (48.2)	189 (56.8)	0.87	0.31
Body mass index, kg/m^2	23.4±3.4	23.7±3.5	23.3±3.4	0.74	0.98
Hypertension	13 (61.9)	39 (69.6)	239 (71.8)	0.71	0.47
Diabetes	4 (19.0)	12 (21.4)	111 (33.3)	>0.99	0.27
Hyperlipidemia	8 (38.1)	25 (44.6)	131 (39.3)	0.80	>0.99
Previous stroke	1 (4.8)	4 (7.1)	66 (19.8)	>0.99	0.15
Ever smoker	11 (52.4)	25 (44.6)	93 (27.9)	0.73	0.032
Previous medication					
Antiplatelet	4 (19.0)	11 (19.6)	132 (39.8)	>0.99	0.097
Anticoagulant	1 (4.8)	3 (5.4)	65 (19.5)	>0.99	0.14
Neurologic status					
Initial NIHSS score	2 [1; 4]	3 [1; 5.5]	5 [2; 14]	0.40	0.005
Discharge NIHSS score	2 [0; 3]	1 [0; 4]	2 [1; 8]	0.73	0.047
Discharge mRS	1 [0; 2]	1 [1; 2]	2 [1; 4]	0.38	0.005
Intravenous thrombolysis	0 (0.0)	3 (5.4)	52 (15.6)	0.56	0.054
Endovascular treatment	0 (0.0)	3 (5.4)	63 (18.9)	0.56	0.033
White blood cells, ×10^9/L	7.690 [6.090; 9.270]	8.425 [6.040; 10.875]	7.480 [6.250; 9.410]	0.48	0.93

Hematocrit, %	40.5 [34.9; 45.1]	40.6 [36.4; 45.0]	40.0 [35.9; 44.4]	0.94	0.74
Platelets, $\times 10^9$/L	234 [207; 276]	234 [195.5; 268.5]	201 [167; 233]	0.75	0.007
Creatinine, mg/dL	0.82 [0.70; 0.99]	0.86 [0.70; 0.99]	0.90 [0.74; 1.12]	0.95	0.22
Fasting glucose, mg/dL	99 [90; 111]	97.5 [85; 106]	100 [87; 124]	0.44	0.60
LDL cholesterol, mg/dL	118 [100; 139]	118 [87.5; 152]	96.5 [71; 125]	>0.99	0.030
PT-INR	0.98 [0.92; 1.01]	0.97 [0.94; 1.01]	1.03 [0.97; 1.11]	0.84	0.007
Fibrinogen, mg/dL	331 [262; 378]	334 [293; 384]	320 [280; 372]	0.35	0.75
hsCRP, mg/dL	0.14 [0.08; 0.47]	0.15 [0.10; 0.56]	0.23 [0.08; 0.81]	0.52	0.23
LVIDd, cm	4.8 [4.6; 5.1]	4.7 [4.5; 5.0]	4.7 [4.4; 5.0]	0.20	0.069
LVIDs, cm	3.0 [2.9; 3.2]	2.9 [2.7; 3.1]	3.0 [2.7; 3.2]	0.26	0.37
IVSd, cm	0.9 [0.9; 1.0]	0.9 [0.8; 1.0]	1.0 [0.9; 1.1]	0.68	0.33
LVPWd, cm	0.9 [0.9; 1.0]	0.9 [0.9; 1.0]	1.0 [0.9; 1.0]	0.32	0.89
LV mass index, g/m^2	92.5 [86.2; 106.8]	89.0 [77.6; 106.2]	93.4 [78.6; 109.0]	0.37	0.73
LV ejection fraction, %	60.5 [57; 63]	61 [57.5; 65]	60 [56; 64]	0.45	0.92
LA size, mm	37.5 [35; 43]	39 [35; 43]	49 [43; 53]	0.76	<0.001
Subclinical valve lesion	1 (4.8)	8 (14.3)	67 (20.1)	0.43	0.093

The data are expressed as numbers (%), mean ± standard deviation, or median [interquartile range].

*p*1, *p*-value between definite APS-stroke and aPL-stroke; *p*2, *p*-value between definite APS-stroke and AF-stroke.

Abbreviations: AF-stroke, atrial fibrillation-related stroke; aPL-stroke, antiphospholipid antibody-related stroke; APS-stroke, antiphospholipid syndrome-related stroke; hsCRP, high-sensitivity C-reactive protein; IVSd, interventricular septal thickness at end-diastole; LDL, low-density lipoprotein; LV, left ventricular; LVIDd, left ventricular internal diameter at end-diastole; LVIDs, left ventricular internal diameter at end-systole; LVPWd, left ventricular posterior wall thickness at end-diastole; mRS, modified Rankin Scale; NIHSS, National Institutes of Health Stroke Scale; PT-INR, prothrombin time-international normalized ratio.

Table S2. Imaging characteristics of the definite APS-stroke, aPL-stroke, and AF-stroke groups

	Definite APS-stroke (n=21)	aPL-stroke (n=56)	AF-stroke (n=333)	*p*1	*p*2
DWI lesion pattern					
Single small lesion	6 (28.6)	17 (30.4)	25 (7.5)	>0.99	0.006
Small scattered lesions in single territory	4 (19.0)	8 (14.3)	44 (13.2)	0.73	0.51
Large territorial lesion	7 (33.3)	15 (26.8)	188 (56.5)	0.78	0.066
Multi-territory lesions	4 (19.0)	16 (28.6)	76 (22.8)	0.58	0.80
Relevant artery occlusion	3 (14.3)	10 (17.9)	182 (54.7)	>0.99	0.001
Intracranial branching vessels	2 (9.5)	3 (5.4)	73 (21.9)	0.61	0.27
Intracranial main vessels	1 (4.8)	6 (10.7)	96 (28.8)	0.67	0.032
Extracranial large vessels	0 (0.0)	1 (1.8)	13 (3.9)	>0.99	>0.99
Total DWI lesion volume, mL	1.6 [0.5; 9.5]	1.6 [0.5; 9.4]	11.3 [2.8; 33.1]	0.89	0.003

The data are expressed as numbers (%) or median [interquartile range].

*p*1, *p*-value between definite APS-stroke and aPL-stroke; *p*2, *p*-value between definite APS-stroke and AF-stroke.

Abbreviations: AF-stroke, atrial fibrillation-related stroke; aPL-stroke, antiphospholipid antibody-related stroke; APS-stroke, antiphospholipid syndrome-related stroke; DWI, diffusion-weighted imaging.

Figure S1. Diffusion-weighted imaging lesion pattern and total lesion volume of definite APS-stroke and AF-stroke patients with a multi-territory lesion

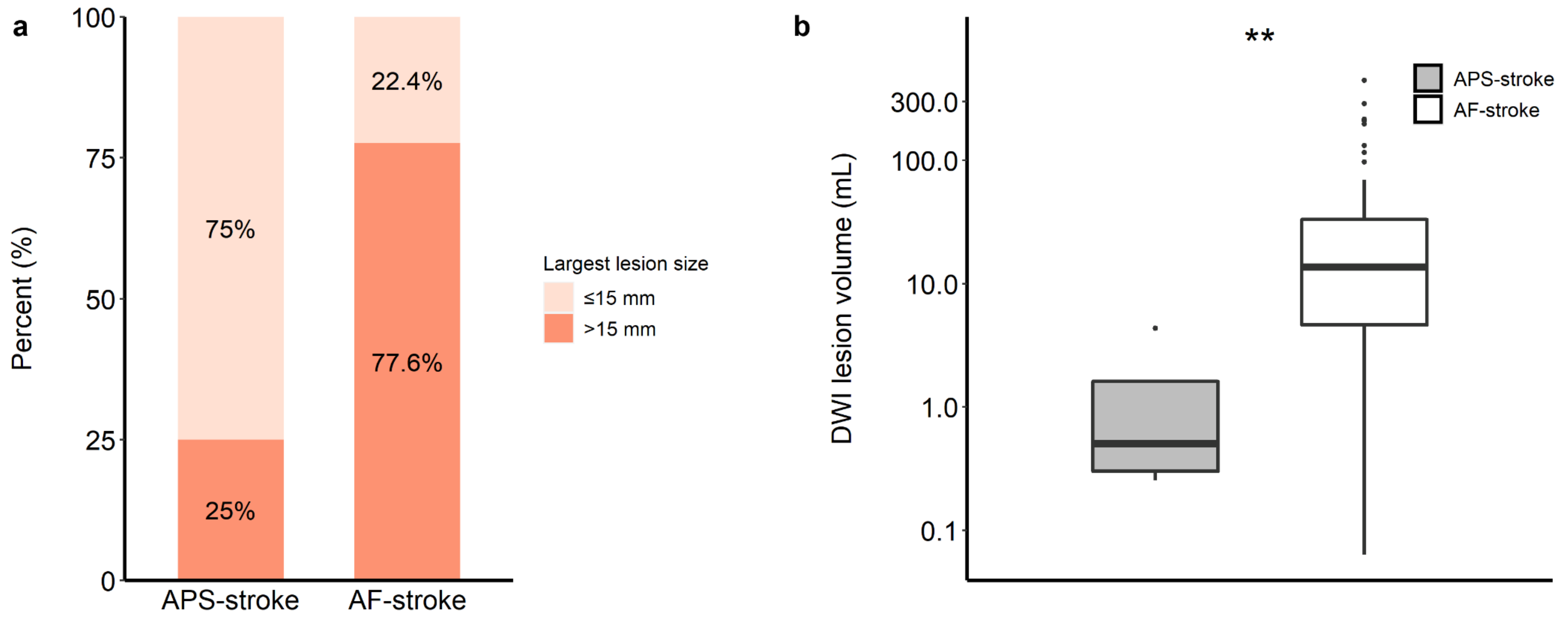

(a) Lesion pattern based on the largest lesion size (≤15 mm or >15 mm) of definite APS- and AF-stroke patients with multi-territory lesions. (b) Total DWI lesion volume of definite APS- and AF-stroke patients with multi-territory lesions. DWI lesion volume is presented on the y-axis as a log scale.

** $p<0.01$. Abbreviations: AF-stroke, atrial fibrillation-related stroke, APS-stroke, antiphospholipid syndrome-related stroke; DWI, diffusion-

weighted imaging.

Table S3. The coexisting potential sources of stroke that were excluded from the analysis in the aPL- and AF-stroke groups.

aPL-stroke	AF-stroke
Large artery atherosclerosis (n=26)	Large artery atherosclerosis (n=10)
Cardioembolic source (n=20)	Cardioembolic source other than AF (n=200)
AF (n=10)	Congestive heart failure (n=42)
Left ventricular akinesia (n=5)	Left ventricular akinesia or significant hypokinesia (n=40)
Prosthetic valve (n=4)	Prosthetic valve (n=39)
PFO or ASD (n=4)	Mitral stenosis (n=26)
Left atrial thrombus (n=1)	Mitral valve prolapse (n=5)
≥2 cardioembolic sources (n=4)	Sick sinus syndrome or complete atrioventricular block (n=25)
Other cause (n=28)	PFO or ASD (n=21)
Cancer (n=13)	Dilated cardiomyopathy (n=12)
Intra/extracranial dissection (n=5)	Hypertrophic cardiomyopathy (n=16)
Moyamoya disease (n=3)	Left atrial thrombus (n=7)
Procedure-related stroke (n=4)	Cardiac amyloidosis (n=1)
Pulmonary AVM (n=2)	≥2 other cardioembolic sources (n=32)
Aortic dissection (n=1)	Other cause (n=3)
≥2 sources other than aPL (n=1)	Intra/extracranial dissection (n=1)
	Moyamoya disease (n=2)
	Positive aPL (n=10)
	≥2 sources other than AF (n=5)

Abbreviations: AF, atrial fibrillation; AF-stroke, atrial fibrillation-related stroke; aPL, antiphospholipid antibody; aPL-stroke, antiphospholipid antibody-related stroke; ASD, atrial septal defect; AVM, arteriovenous malformation; PFO, patent foramen ovale.

Figure S2. Representative examples of the diffusion-weighted imaging lesion pattern

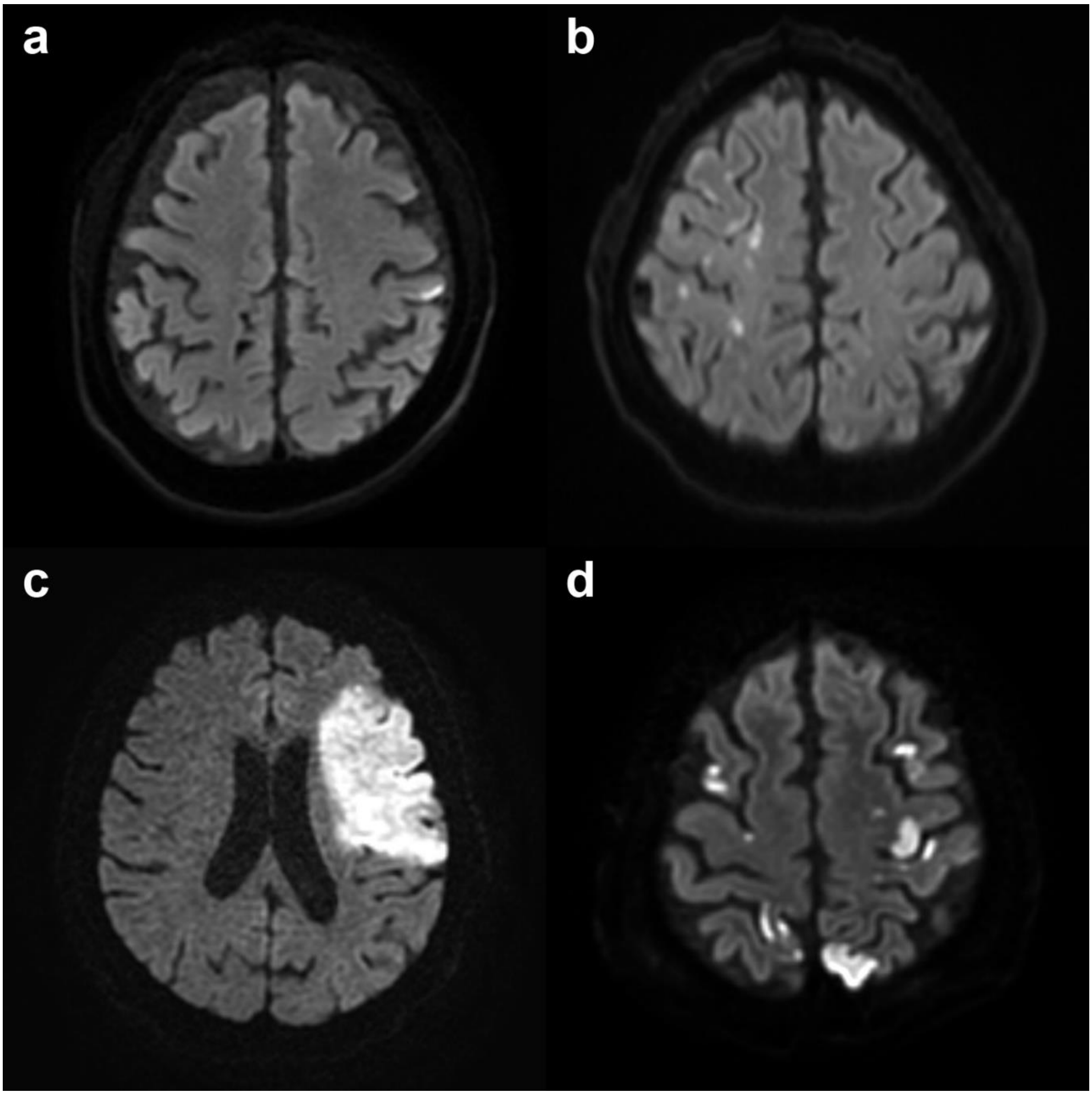

(a) Single small lesion, a solitary lesion ≤15 mm; (b) small scattered lesion in a single territory, multiple scattered lesions with the largest lesion size ≤15 mm; (c) large territorial lesion, lesion >15 mm involving a single vascular territory; and (d) multi-territory lesion, multiple lesions involving multiple vascular territories.

Figure S3. Representative example of semiautomated segmentation of infarcted areas on diffusion-weighted imaging.

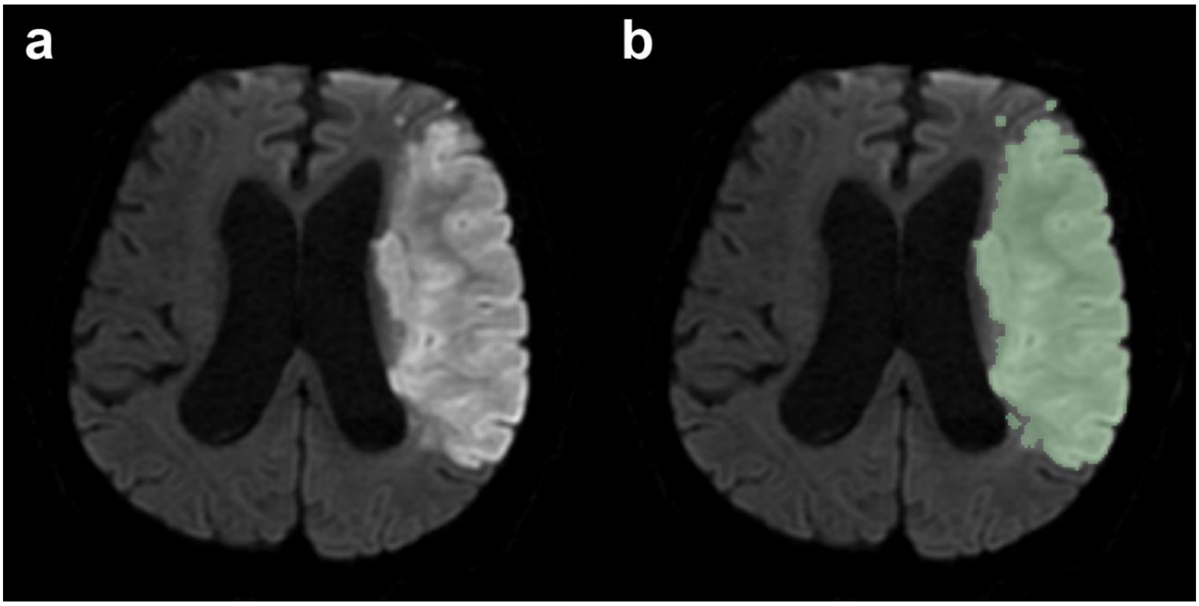

(a) The infarcted region shows a high signal intensity in the diffusion-weighted image of patients with left middle cerebral artery territory infarction. (b) The corresponding segmentation of the infarcted area for calculation of the infarct volume, based on a semiautomated method using 3D slicer software.

Figure S4. Representative examples of patterns of relevant artery occlusion

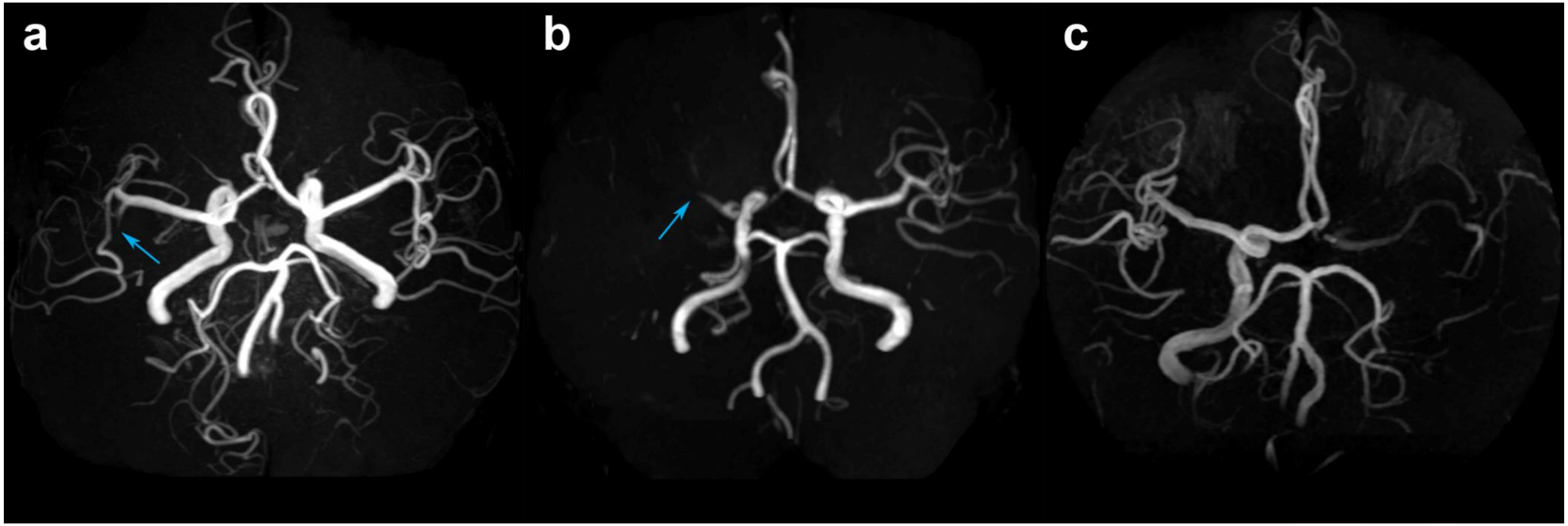

(a) Intracranial branch vessel occlusion, occlusion of the ACA, PCA, M2 or distal segments of the MCA, or SCA; (b) intracranial main vessel occlusion, occlusion of the distal ICA, M1 segment of the MCA, distal VA, or BA; and (c) extracranial large vessel occlusion, occlusion of the CCA, proximal ICA, or proximal VA.

Abbreviations: ACA, anterior cerebral artery; BA, basilar artery; CCA, common carotid artery; ICA, internal carotid artery; MCA, middle cerebral artery; PCA, posterior cerebral artery; SCA, superior cerebellar artery; VA, vertebral artery.
